# Risk Factors and Clinical Outcomes for Patients With *Acinetobacter baumannii* Bacteremia

**DOI:** 10.1097/MD.0000000000002943

**Published:** 2016-03-07

**Authors:** Zhenyang Gu, Yuliang Han, Taojiang Meng, Shasha Zhao, Xiaoli Zhao, Chunji Gao, Wenrong Huang

**Affiliations:** From the Department of Hematology, Chinese People's Liberation Army General Hospital, Haidian District (ZG, SZ, XZ, CG, WH), Department of Neurology, Chinese People's Liberation Army 305 Hospital, Xicheng District, Beijing (YH), Sanya Sanitorium of Chinese People's Armed Police Forces (TM), and Department of Hematology, Hainan Branch of Chinese People's Liberation Army General Hospital (TM, WH), Sanya City, Hainan, China.

## Abstract

*Acinetobacter (A.) baumannii*, an opportunistic nosocomial pathogen that can cause significant morbidity and mortality, has emerged as a worldwide problem. This study aimed to analyze the clinical features and outcomes of patients with *A. baumannii* bacteremia and determine the factors influencing survival by using 14-day mortality as the primary endpoint.

A 6-year retrospective study of 122 cases with monomicrobial *A. baumannii* bacteremia was conducted in Chinese People's Liberation Army (PLA) General Hospital from January 2008 to April 2014. Predictors of 14-day mortality were identified by logistic regression analysis.

The overall 14-day mortality rate was 40.2% (49 of 122 patients). Multivariable analysis revealed that independent predictors of 14-day mortality included severity of illness defined by Pitt Bacteremia Score (PBS) (odds ratio [OR], 0.46; 95% confidence interval [CI], 0.340–0.619; *P* < 0.001), neutropenia (OR, 18.02; 95% CI, 1.667–194.67; *P* = 0.017), and malignancy (OR, 4.63; 95% CI, 1.292–16.588; *P* = 0.019). The effect of malignancy was influenced by neutropenia (OR for interaction term, 1.60; 95% CI, 1.15–2.22; *P* = 0.005). A subgroup analysis revealed that 14-day mortality rate for patients with underlying hematological malignancies and solid tumors was 75% (12/16) and 40% (12/30), respectively. Survival analysis revealed that mortality in patients with hematological malignancies was higher than that in patients with solid tumors (*P* = 0.032).

The outcomes of patients with *A. baumannii* bacteremia were related to PBS, neutropenia, and malignancy. Compared with solid tumors, patients with hematological malignancies had a higher mortality in the setting of *A. baumannii* bacteremia.

## INTRODUCTION

*Acinetobacter (A.) baumannii* is one of the most common, gram-negative pathogens causing bacteremia.^1^ Bacteremia is an important and prevalent cause of patient mortality and the overall mortality in patients with *A. baumannii* bacteremia ranges widely from 29% to 63%.^1–3^ Furthermore, due to increasing exposure to antibiotics, multidrug resistance (MDR) and carbapenem resistance rates have been predictably increasing these years.^4,5^ With limited treatment options, infections caused by multidrug resistant and carbapenem resistant *A. baumannii* might result in higher mortality.

Several studies have investigated predictors of mortality in patients with *A. baumannii* bacteremia. Risk factors independently associated with mortality include drug resistance, severity of illness, appropriate antimicrobial therapy, malignancy, and other comorbidities such as immunosuppression.^6–9^ However, previous studies suffered from different limitations which make it difficult to draw definitive conclusions, such as failure to distinguish between *A. baumannii* colonization and infection, inappropriate clinical endpoints, failure to adjust for confounders such as severity of illness and other comorbid conditions.

To further understand risk factors influencing survival in patients with *A. baumannii* bacteremia, this retrospective study was conducted to analyze the clinical features and outcomes of patients with *A. baumannii* bacteremia and determine factors influencing survival by using 14-day mortality as the primary endpoint.

## METHODS

### Study Design and Population

A retrospective study was conducted to investigate risk factors influencing survival in adult patients (≥18 years old) with *A. baumannii* bacteremia consecutively admitted to the Chinese People's Liberation Army (PLA) General Hospital, a 3000-bed, tertiary care teaching hospital in Beijing, China, from January 2008 to April 2014. Charts were reviewed for all patients with ≥1 *A. baumannii* bacteremic episodes who had symptoms and signs of infection. For patients with ≥2 bacteremic episodes, only the first episode was included. Patients with polymicrobial bacteremia and those with incomplete medical records were excluded. Patient records/information was anonymized and deidentified before analysis. This study was reviewed and approved by the Medical Ethics Committee of PLA General Hospital.

### Organism Identification and Susceptibility Classification

Blood specimens drawn at the bedside under sterile conditions were processed in an automated blood culture machine. Identification of the isolates to the level of the *A. baumannii* complex and antimicrobial susceptibility tests were completed using a Vitek II system (bioMerieux, Marcy-lEtoile, France). Antimicrobial susceptibility was performed by disk diffusion method and results were interpreted according to Clinical Laboratory Standards Institute criteria. Intermediate resistance was regarded as resistance in our study. MDR was defined as resistance to ≥3 of the following classes of antimicrobials: antipseudomonal cephalosporins, antipseudomonal carbapenems, ampicillin-sulbactam, fluoroquinolones, and aminoglycosides.^10^ Carbapenem resistance was defined as resistance to imipenem and meropenem.

### Data Collection

Demographic characteristics of patients included age, gender, dates of admission and discharge, duration of hospital stay before development of bacteremia. We also collected patient medical comorbidities (diabetes, hepatic dysfunction, renal dysfunction, underlying malignancy, coronary arterial disease, congestive heart failure, and chronic obstructive pulmonary disease), recent surgery (performed within 4 weeks before the onset of bacteremia), history of immunosuppression (ie, corticosteroids or chemotherapy within the previous 6 months or HIV-positive status), absolute neutrophil count, severity of illness defined by the Pitt Bacteremia Score (PBS) within 24 hours before bacteremia onset, the presence of a ventilator, central venous catheters, a nasogastric tube, or a Foley catheter at the time of bacteremia onset, antimicrobial susceptibility, time of receipt, dose and route of therapy with individual antimicrobial drugs, the sources of bacteremia and mortality. The PBS system was an efficient index to determine the severity of sepsis in critically ill patients, to compare patient outcomes, and more importantly, to predict clinical outcomes and guide physicians in the management of patients.

### Definitions

The onset of bacteremia was defined at the time when the blood specimens that eventually yielded *A. baumannii* was drawn. Neutropenia was defined as an absolute neutrophil count <500 cells/mm^3^. Renal impairment was defined as an estimated glomerular filtration rate <60 mL/min/1.73 m^2^. Antimicrobial therapy was defined to be “appropriate” if the antibiotics, which were administered within 48 hours after the onset of bacteremia, included at least 1 antibiotic that was active in vitro and when the dosage, duration, and route of administration were in accordance with current medical standards. Antimicrobial therapy which did not meet this definition was considered “inappropriate.” Source of bacteremia was recorded as documented or indirectly inferred from the infection diagnosis on the day of bacteremia according to the definitions of the Centers for Disease Control and Prevention.^11^ Given 30 days is too long to interpret the cause of mortality for critically ill patients and 7 days is too short a time to witness a response to treatment,^2,3,9^ we chose all-cause 14-day mortality after the onset of *A. baumannii* bacteremia as the primary outcome measure.

### Statistical Analysis

Continuous variables were presented by median values and interquartile ranges (IQRs) and calculated with the Student *t* test or Mann–Whitney *U* test as appropriate. Categorical variables presented by percentage were calculated with Pearson Chi-square test with Yate correction or Fisher exact test. Logistic regression models were used to explore independent risk factors for 14-day mortality. All biologically plausible variables with a *P*-value <0.10 in the univariable analysis were entered into a multivariable backward logistic regression analyses to assess their relationship with mortality. The interaction analysis between the PBS and the covariates was chosen using an interaction term. Kaplan–Meier survival analysis were performed to determine the time to mortality, which was defined as the interval between bacteremia onset and death. All the analyses were performed using Statistical Package for the Social Sciences (SPSS) software version 19.0. A *P* < 0.05 was considered to be statistically significant.

## RESULTS

A total of 142 adult patients with ≥1 blood culture positive for the *A. baumannii* complex were identified during our study period. After excluding 14 patients with incomplete clinical records and 6 patients with polymicrobial bacteremia, 122 patient bacteremic episodes were included in the final analysis. Forty-nine patients (40.2%, 49/122) died within 14 days after the onset of monomicrobial *A. baumannii* bacteremia. Sixty-nine patients (55.6%, 69/122) were found to acquire their *A. baumannii* bacteremia in the ICU.

The demographic and clinical characteristics of the 122 patients with *A. baumannii* bacteremia stratified by 14-day mortality are summarized in Table 1. there weren’t other differences in demographic characteristics and comorbid conditions besides those shown in Table 1. The group of patients who died within 14 days after the onset of bacteremia were significantly more likely to have malignancy, neutropenia, receive immunosuppressive therapy, have higher PBS, and higher rates of nasogastric tubes and ventilator use. The percentage of MDR of all patients was over 84.4%(103/122), and the percentage among the group who died reached up to 98%, which is significantly higher that of the alive group (75.3%). The blood isolates from the group who died also had a significantly greater rate of resistance to carbapenems than the control group (98.0% vs 76.7%, *P* < 0.001). No significant differences were found in resistance to antipseudomonal cephalosporins, ampicillin-sulbactam, fluoroquinolones, aminoglycosides, and piperacillin-tazobactam between isolates from these 2 groups (data not shown). The group of patients who died within 14 days tended to be less likely to received appropriate antimicrobial therapy than the alive group (4.1% vs 20.5%, *P* = 0.01). There were also no significant difference in the percentage of using sulbactam containing regimens and the source of bacteremia between these 2 groups.

**TABLE 1:**
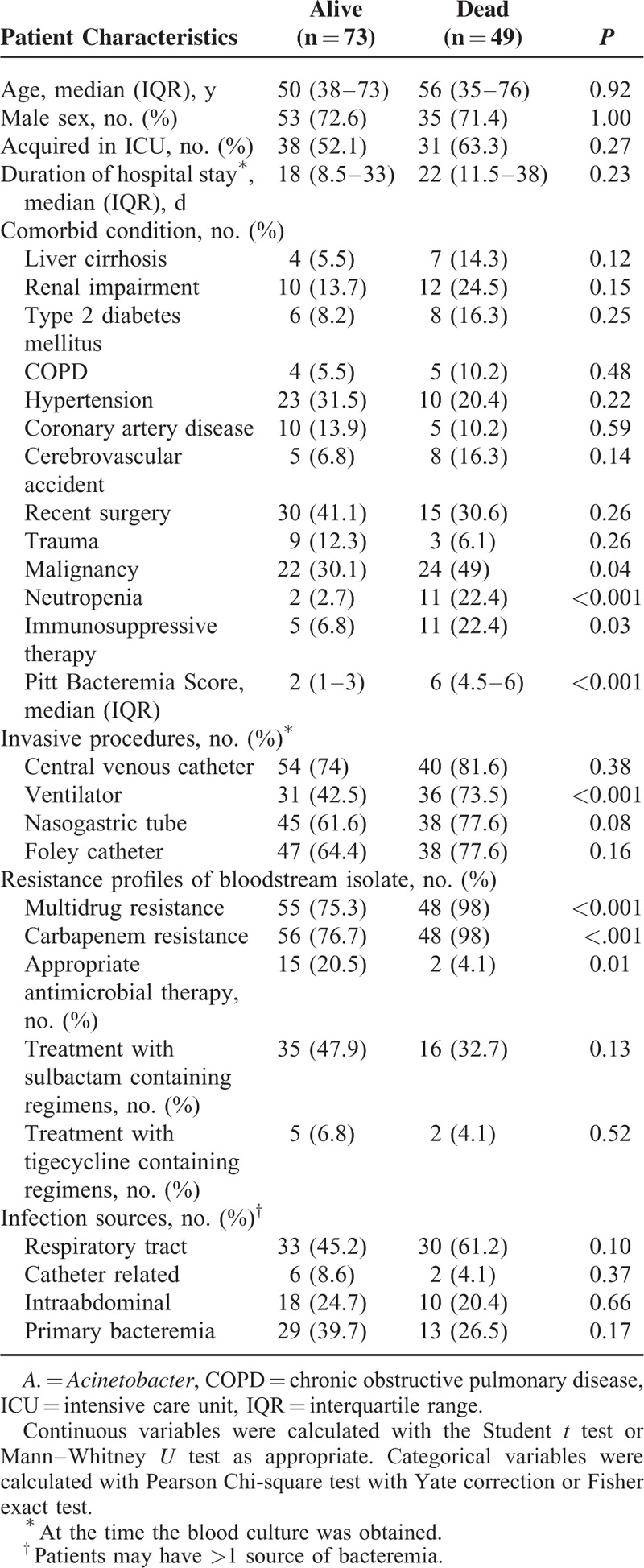
Demographic and Clinical Characteristics of Patients With *A. baumannii* Bacteremia Stratified by 14-Day Mortality

Logistic regression analysis results are shown in Table 2. Independent predictors of 14-day mortality were revealed to be PBS (odds ratio [OR], 0.46; 95% confidence interval [CI], 0.340–.619; *P* < 0.001), neutropenia (OR, 18.02; 95% CI, 1.667–194.67; *P* = 0.017), and malignancy (OR, 4.63; 95% CI, 1.292–16.588; *P* = 0.019) in multivariable analysis. Whereas the use of appropriate antimicrobial therapy or drug resistance was not found to be associated with the mortality in multivariable analysis. Similar results were also obtained using the multivariate Cox regression model (data not shown).

**TABLE 2:**
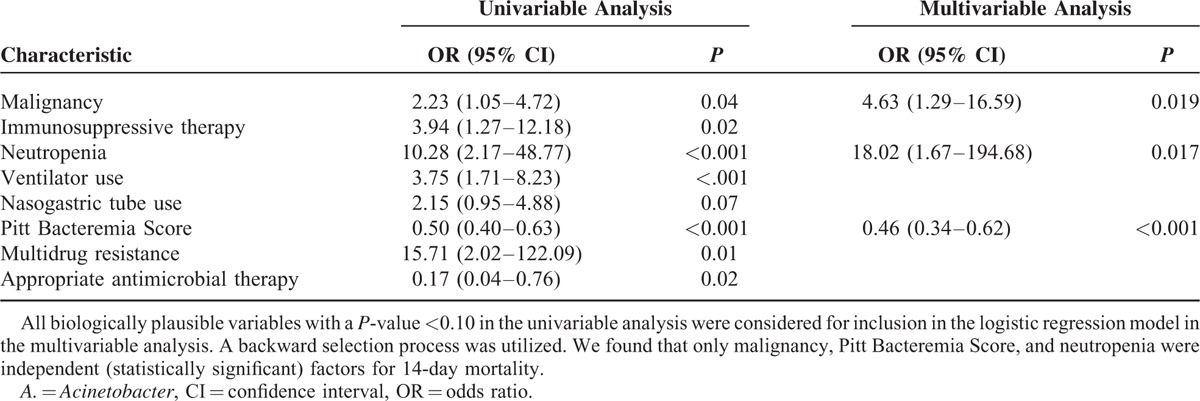
Logistic Regression Analysis of Predictors for 14-Day Mortality Among Patients With *A. baumannii* Bacteremia

**FIGURE 1:**
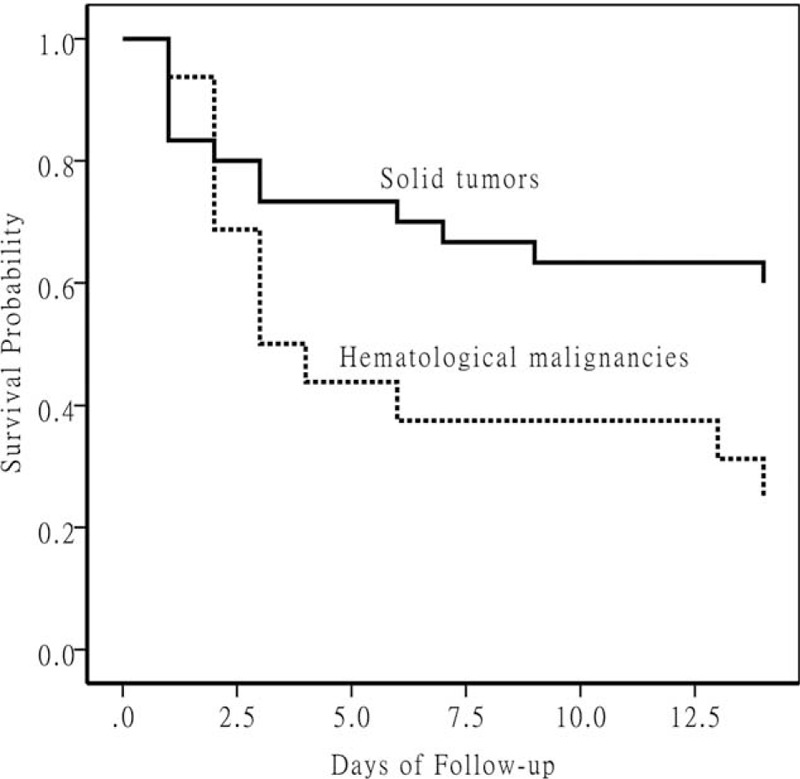
Kaplan–Meier survival curves at 14 days after *Acinetobacter baumannii* bacteremia onset for patients with hematological malignancies versus patients with solid tumors (*P* = 0.032).

To further investigate the relationship between malignancy and neutropenia, interactions between these 2 variables were added to the logistic regression model. The interaction term was statistically significant (OR for interaction term, 1.60; 95% CI, 1.15–2.22; *P* = 0.005). Through further investigation we found that if the patients with malignancy were divided into patients with hematological malignancies and patients with solid tumors, almost all the patients with neutropenia (11/13) suffered from hematological malignancies (13 patients with neutropenia: 11 patients with hematological malignancies, 1 patient with solid tumors, and 1 from nontumor patient). To clarify the impact of different underlying tumors on mortality, Kaplan–Meier survival curves were developed for patients with hematological malignancies and patients with solid tumors (*P* = 0.032, Figure 1). A comparison of the demographic and clinical characteristics between patients with hematological malignancies (16 patients) and patients with solid tumors (30 patients) revealed that patients with hematological malignancies were younger and more likely to have had neutropenia and immunosuppressive therapy, but less likely to have had recent surgical procedures (Table 3). There was no significant difference in sex, PBS, drug resistance, and appropriate antimicrobial therapy (Table 3). Further classification of the 16 patients with hematologic malignancies revealed that this group was composed of 7 patients with lymphoma, 5 patients with acute myeloid leukemia, 3 patients with acute lymphoblastic leukemia, and 1 patient with chronic myeloid leukemia.

**TABLE 3:**
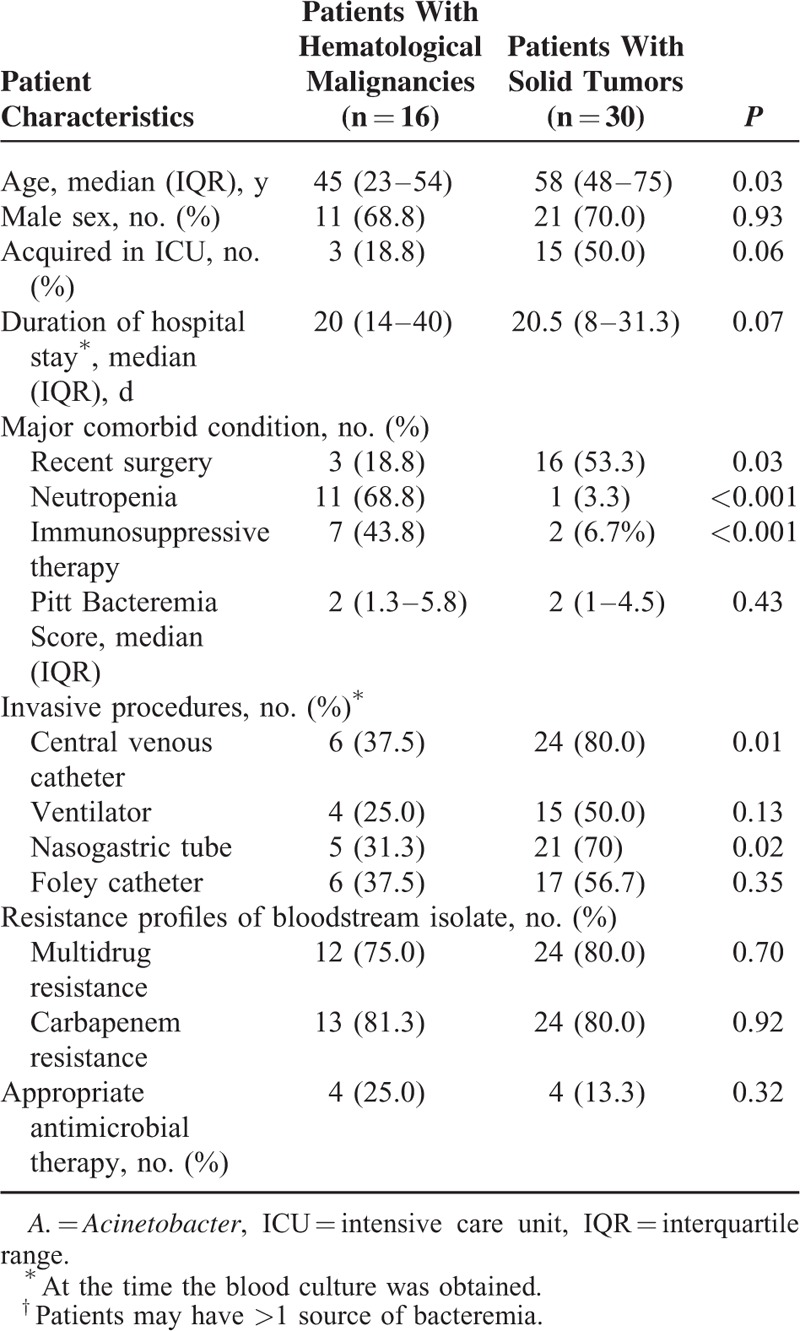
Demographic and Clinical Characteristics of *A. baumannii* Bacteremia Patients With Different Underlying Tumors: Solid Tumors Versus Hematological Malignancies

## DISCUSSION

We attempted to identify risk factors influencing early death in patients with monomicrobial *A. baumannii* bacteremia through this retrospective study, which consisted of 122 patients admitted to a large teaching hospital in China. Severity of illness defined by PBS, neutropenia, and malignancy were found to be independent factors for 14-day mortality. Whereas the use of appropriate antimicrobial therapy or drug resistance was not found to be associated with the mortality in multivariable analysis. Further analysis also revealed that for patients with *A. baumannii* bacteremia, patients who suffered hematological malignancies had a higher mortality than that of those who suffered solid tumors.

In our study, the mortality rate is 40.2%, which is comparable to the range of 29% to 63% reported by others.^1–3^ Thus, crude mortality rate differed significantly between patients in general wards and ICUs, and patients infected by *A. baumannii* with various drug resistance profiles.^12^ In this study, a total of 69 patients (55.6%, 69/122) acquired *A. baumannii* bacteremia in the ICU. And the percentage of MDR and carbapenems resistance was 84.4%(103/122) and 85.2%(105/122), respectively.

Like others, both drug resistance and the use of appropriate antimicrobial therapy were significantly different in bivariable analysis.^2,13,14^ But neither of these 2 variables reached difference as an independent risk factor for mortality in subsequent multivariable analysis. It is contrary to previous argument about the association of high mortality with antibiotic resistance in patients with *A. baumannii* bacteremia.^3,9^ Patients with short life expectancies have been proved to be unable to benefit significantly from appropriate antimicrobial therapy.^15^ In our study, 55.6% of the patients acquired *A. baumannii* bacteremia in the ICUs. So these results can at least be partly explained by the fact that our study population might contain many severely ill patients. Nevertheless, the rates of MDR and carbapenems resistance both excessed 84% in our study. And together with the fact that most patients have no access to tigecycline and colistin during our study period, only 13.9% (17/122) of patients received appropriate antimicrobial therapy. These results may be also confounded by the small sample size of this study.

In this study, we also analyzed the effectiveness of sulbactam containing regimens for *A. baumannii* bacteremia. Unfortunately, it was found to be not significantly associated with decreased mortality both in bivariable analysis and multivariable analysis. This was consistent with the results of a recent systematic review.^16^ With the increasing rate of drug resistance of *A. baumannii*, new antimicrobial agents are urgently needed. Tigecycline has been proved to be promising in the treatment of *A. baumannii* infections.^17–19^ However, tigecyclines were not commercially available until 2013 in China. In our study, 7 patients were treated with tigecycline and only 2 of them died. This supports the need for further clinical studies investigating the role of tigecycline in patients with *A. baumannii* bacteremia.

In our study, severity of illness defined by PBS, neutropenia, and malignancy were all found to be independent factors for 14-day mortality. All these variables have been reported as independent predictors of death in patients with *A. baumannii* bacteremia.^9,14,20–23^ Nevertheless, crude mortality can be caused by patients’ underlying diseases as well as infections, which tend to be both frequent and severe in such patients.^24,25^ The point that outcomes of patients with infection correlated more closely with their underlying illness than with other factors was reported in an early study of *A. baumannii* bacteremia.^26^ Case–control studies also demonstrate that the presence of *A. baumannii* bacteremia did not correlate with a significantly increased mortality rate in critically ill patients.^25,27^ Both of them concluded that underlying illnesses seemed to play a more important role than the infection itself as the cause of death. PBS, neutropenia, and malignancy can reflect different aspects of underlying diseases. So our findings were not unexpected.

Our study demonstrated that underlying malignancy was an independent factor for mortality in patients with *A. baumannii* bacteremia, which concurred with previous results.^9,22^ Thus our investigation further pointed out that for patients with *A. baumannii* bacteremia, patients who suffered hematological malignancies tended to have a higher mortality than that of patients who suffered solid tumors. And patients with hematological malignancies also tended to be more likely to experience neutropenia and receive immunosuppressive therapy. New chemotherapeutic approaches with solid tumors have substantially decreased neutropenia-associated toxicity.^28^ However, patients with hematological malignancies still receive conventional chemotherapy and intensive immunosuppressive therapy. So neutropenia and immunosuppression was prevalent in these kind of patients.^29,30^

There were some inherent limitations in our study. First, this was a retrospective study with a relatively small number of patients. Second, patients with all *A. baumannii* complex bacteremia were all included in this study based on a single tertiary care medical center. Nevertheless, this study was strengthened by the exclusion of subjects deemed to be colonized and patients with polymicrobial bacteremia, adjustment for confounders and a well-defined end point of 14-day mortality.

Our study revealed that the independent risk factors influencing 14-day mortality for patients with *A. baumannii* bacteremia were severity of illness defined by PBS, neutropenia, and underlying malignancy, which implied that underlying illnesses seemed to play a more important role than the infection itself as the cause of death. Compared with solid tumors, underlying hematological malignancies were associated with higher mortality for patients with *A. baumannii* bacteremia.
